# Circulating type I collagen pro-α1 chain is inversely associated with the presence of coronary atherosclerosis in a Swedish middle-aged population

**DOI:** 10.1038/s41598-026-45736-2

**Published:** 2026-03-26

**Authors:** Filip Hammaréus, Lennart Nilsson, Rosanna W.S. Chung, Fredrik H Nyström, Carl Johan Östgren, Lena Jonasson

**Affiliations:** 1 Department of Health, Medicine and Caring Sciences, Linköping University, Linköping, SE-58183 Sweden; 2 Department of Cardiology, Linköping University Hospital, Region Östergötland, Linköping, Sweden; 3 Department of Cardiology, Ryhov hospital, Region Jönköpings län, Jönköping, Sweden; 4 Centre for Medical Image Science and Visualization, Linköping University, Linköping, SE-58183 Sweden

**Keywords:** Collagen type I, Biomarker, Extracellular matrix, Coronary artery disease, Computed tomography angiography, Atherosclerosis, Biomarkers, Cardiology, Diseases

## Abstract

The turnover of type I collagen plays an important role in atherogenesis and atherothrombosis. The pro-α1(I) chain of type I collagen (COL1α1) has emerged as a novel circulating biomarker associated with coronary artery disease (CAD) events. This study aimed to investigate the association between circulating COL1α1 and coronary atherosclerosis in a middle-aged general population. Participants (*n* = 1 078) were randomly recruited from the Linköping branch of the Swedish Cardiopulmonary Bioimage Study (SCAPIS) comprising an equal number of men and women 50–64 years old. Coronary artery calcium scores were calculated from non-contrast computed tomography (CT) while coronary stenosis, presence of plaques and segmental involvement scores were derived from coronary CT angiography. COL1α1, interleukin-6 and matrix metalloproteinase-9 were quantified in plasma. Binary logistic regression models were used. Participants with low COL1α1 levels exhibited a more unfavorable cardiovascular risk profile. There was an inverse association between COL1α1 and all measures of coronary atherosclerosis before adjustment (OR_range_=0.50–0.73, *p* < 0.05 for all). In two multivariable models adjusting for potential confounders and CAD risk factors, an association with coronary stenosis ≥ 50% (OR = 0.61 and 0.59, respectively) and non-calcified atherosclerotic plaques (OR = 0.62 for both) remained (*p* < 0.05 for all). The findings shed further light on COL1α1 as a potential CAD biomarker.

**Supplementary Information:**

The online version contains supplementary material available at 10.1038/s41598-026-45736-2.

## Introduction

Atherosclerosis can go unnoticed for decades before clinical manifestation^1^. As recently shown in a Swedish population-based study, around 40% of middle-aged (50–64 years) individuals had detectable coronary atherosclerosis on coronary computed tomography angiography (CCTA)^2^. The synthesis of collagen plays a pivotal role in the initial formation of an atherosclerotic plaque and hence also in subclinical coronary artery disease (CAD). However, this may be followed by a shift promoting collagen degradation with the ensuing risk of plaque rupture, thrombosis and CAD events^3,4^. Type I collagen, which constitutes around two-thirds of the plaque collagen content, is initially assembled by two pro-α1(I) chains (COL1α1) and one pro-α2(I) chain (COL1α2)^4,7^ . As recently reported^5,6^, there was an inverse relationship between plasma levels of COL1α1 and future CAD events that remained intact even after adjustments for established cardiovascular risk factors. As further shown in these studies, COL1α1 was strongly correlated to a marker of type I collagen synthesis (PRO-C1), but neither to markers of inflammation nor collagen degradation. The findings shed light on the role of collagen synthesis in atherosclerosis, possibly independent of inflammation and matrix degradation; the latter being processes that have been extensively investigated previously^7–13^.

COL1α1 may thus represent a novel biomarker for cardiovascular risk prediction. However, it is unknown whether this relationship is mediated through the development of atherosclerosis or other mechanisms, such as interaction with coagulation or myocardial vulnerability^14^. The major aim of the present study was to elucidate whether COL1α1 in plasma was associated with the presence and extent of coronary atherosclerosis as assessed by coronary artery calcium scores (CACS) and CCTA in a subcohort of the Swedish Cardiopulmonary Bioimage Study (SCAPIS) population. In addition, we assessed the associations between COL1α1, clinical characteristics and markers of systemic inflammation and matrix degradation.

## Methods

### Study population

SCAPIS has been described in detail previously^2,15,16,24^. In brief, SCAPIS is a nation-wide population-based prospective cohort study that randomly recruited Swedish citizens aged 50–64 (*n* = 30 154). The present study is based on a subcohort (“SCAPIS Leukocyte”) that randomly included 1 078 subjects (543 men and 535 women) from the Linköping site of SCAPIS (*n* = 5 058) with the aim of exploring coronary atherosclerosis through flow cytometry-based immune profiling and biomarker quantification. The original SCAPIS protocol had no exclusion criteria except the inability to understand written and spoken Swedish for informed consent; no additional criteria were applied for the subcohort^15,17^. At study inclusion, blood pressure was measured together with anthropometric measures. Data on smoking status, diabetes, and history of cardiovascular disease along with intake of anti-hypertensive and lipid-lowering medication in the last two weeks was gathered through detailed questionnaires on health and lifestyle. Venous blood samples were collected after an overnight fast for at least 8 h. Blood lipids, plasma glucose, Hemoglobin A1C (HbA1c), high-sensitivity C-reactive protein (CRP) and creatinine were analyzed immediately in the hospital’s core laboratory while plasma was stored at -80 °C for future use. Creatinine levels were used to estimate glomerular filtration rate using the revised Lund-Malmö method^18^. All participants signed a detailed informed consent, and the study was conducted in accordance with the Declaration of Helsinki. SCAPIS has been approved by the ethical review board in Umeå (number 2010-228-31 M) and a separate ethical application was approved for this sub-study by the ethical review board in Linköping (Dnr 2021-06248-02).

### Coronary atherosclerosis

As previously reported, participants were examined by a computed tomography (CT) scan also including a CCTA protocol and systematically assessed for coronary atherosclerosis^2,16^. CACS was calculated according to international standards on the non-contrast images and categorized as moderate ( ≥100) and high ( ≥400). Presence of any stenosis in any of the main coronary branches (left coronary artery, left anterior descending artery or right coronary artery) was classified into stenosis below 50% or above 50%. In case the calcium content of a coronary artery segment was severe and affected the quantification of stenosis levels, it was classified as having a calcium bloom artefact. Similar to Bergström et al.^2^, any calcium blooming detected in a segment of the coronary branch was counted as a stenosis below 50%. The presence of coronary plaques (calcified or non-calcified) in any of the coronary segments in the 18 coronary segment model on the contrast-enhanced images were also assessed^2^. A segmental involvement score (SIS) was calculated as described by Bergström et al.^16^. SIS is a scoring system where every coronary segment affected by atherosclerosis, irrespective of stenosis degree and including calcium blooming, generates one point. For CACS, data were excluded from analysis due to CT not being performed (*n* = 5), previous coronary stent (*n* = 14), or other technical reasons (*n* = 9). For CCTA, apart from missing CT images, data were excluded if no CCTA-image was captured due to contrast allergy, reduced kidney function, intravenous access failure or non-consent (*n* = 50) or if there was a technical failure in the four most proximal coronary segments (*n* = 19) as described previously^2^.

### Biomarker measurements

COL1α1 in Lithium Heparin plasma was quantified using the Magnetic Luminex^®^ Assay (R&D Systems Inc., Minneapolis, MN, USA). Matrix metalloproteinase-9 (MMP-9) was also analyzed in the same assay as a marker of matrix degradation^8^. Samples were analyzed on a Flexmap 3D analyzer (Luminex corp, Hertogenbosch, Netherlands) platform using the Bioplex manager software (Biorad laboratories, California, USA). The analysis process was performed according to the manufacturer’s instructions and has been described elsewhere^19^. On each assay plate, 7 duplicates of the standard sample, one duplicate of the blank sample, and two different internal control plasma samples were used. No sample was outside the quantification range. Inter-assay coefficients of variability (CV%) values were 11.1%/6.3% (first control) and 16.4%/5.1% (second control) for COL1α1 and MMP-9, respectively. The Meso Scale Discovery (MSD) platform (Rockville, Maryland, USA), an electrochemiluminescence assay, was used to measure Lithium Heparin plasma concentrations of the cytokine interleukin-6 (IL-6), a marker of systemic inflammation^11^. The inter-assay coefficient of variation was 22.8%. The IL-6 analysis was made by SciLifeLab, a Clinical biomarkers facility in Uppsala, Sweden.

### Statistical analysis

The distribution of normality was tested for continuous variables using the Kolmogorov-Smirnov analysis. Data are shown as median (interquartile range) for variables that were not normally distributed and were analyzed using the Mann-Whitney U test for group comparison of continuous variables. Data with a normal distribution are presented as mean ± standard deviation and were analyzed using Student´s t-test. For categorical variables, the Chi-squared test was used. Spearman correlation analyses were performed where a correlation coefficient > 0.2 generally considered a “poor-to-fair strength” was considered a minimum for clinical relevancy^20^. To assess the association between COL1α1 and coronary atherosclerosis, logistic regression models were used. Model 1 was a crude model including only COL1α1. Model 2 also included variables with correlation coefficients > 0.2, thus being potential confounders. Lastly, model 3 included established cardiovascular risk factors available in the dataset (sex, waist-hip ratio, age, smoking, use of antihypertensive and lipid-lowering medications, diabetes, HbA1c and CRP)^10,13,21^. Variables were included in a stepwise manner. COL1α1 levels were analyzed after being log-transformed with the base of 2 in the regression models. A 2-tailed *p* ≤ 0.05 was used as a cut-off for statistical significance. The SPSS Statistics software (IBM Corporation, Armonk, New York), version 29, was used for statistical analysis.

## Results

### Participant characteristics and their associations with COL1α1 levels

Baseline characteristics in total and stratified groups according to COL1α1 levels (below or above median), are presented in Table 1. Female sex was more prevalent in the high-COL1α1 group (62% vs. 38%). In the low-COL1α1 group, participants had higher waist-hip ratio, higher systolic blood pressure, and higher levels of glucose, HbA1c and triglycerides while having lower levels of high-density lipoprotein (HDL) cholesterol. This group also used anti-hypertensive and lipid-lowering medication to a larger extent. Also, inflammatory markers (CRP and IL-6) were higher while MMP-9 was lower in the low-COL1α1 group. The glomerular filtration rate was similar between groups. Comparisons were also performed sex-stratified due to the skewness between groups. Amongst the female subjects, differences remained significant regarding waist-hip ratio, diabetes and the use of anti-hypertensive and lipid-lowering medication. The levels of triglycerides, glucose, IL-6, and CRP remained higher while HDL cholesterol and MMP-9 was lower in the low-COL1α1 group compared to the high-COL1α1 group (Supplementary table S1). In the male group, waist-hip ratio and the prevalence of diabetes differences remained between groups. The levels of triglycerides and glucose remained elevated in the low-COL1α1 group (Supplementary table S2). In correlation analysis for the whole cohort, COL1α1 was positively associated with the female sex (*r* = 0.31, *p* < 0.001) and HDL (*r* = 0.26, *p* < 0.001) while negatively associated with waist-hip ratio (*r*=-0.31, *p* < 0.001), glucose (*r*=-0.26, *p* < 0.001), and triglycerides (*r*=-0.22, *p* < 0.001). There were weak but significant, inverse correlations to the inflammatory markers CRP and IL-6 (*r*=-0.10, *p* = 0.002 and *r*=-0.06, *p* = 0.05, respectively) while MMP-9 showcased a positive correlation to COL1α1 (*r* = 0.08, *p* = 0.01), see supplementary Fig. 1–3.

**Table 1:** Characteristics of the study population in total and divided by high or low COL1α1 levels (below or above median level).

COL1α1 (pg/mL)	Total (*n* = 1078)	Low COL1α1 (*n* = 539)	High COL1α1 (*n* = 539)	*p*
	6070 (4430)	4250 (1880)	8680 (4010)	< 0.001
*Characteristics*				
Age, years	57.2 (7.5)	57.2 (7.7)	57.1 (7.2)	0.1
Women, n (%)	535 (49)	199 (38)	330 (62)	< 0.001
Current smoker, n (%) ^a^	75 (6.9)	41 (7.7)	33 (6.2)	0.3
Waist-hip ratio	0.91 (0.13)	0.93 (0.12)	0.88 (0.12)	< 0.001
Diabetes, n (%)^ a^	86 (8.0)	61 (12)	22 (4.1)	< 0.001
Anti-hypertensive medication, n (%) ^a^	190 (18)	115 (22)	71 (13)	< 0.001
Office SBP, mmHg	129 (23)	131 (23	128 (24)	0.007
Office DBP, mmHg	82 (14)	83 (14)	81 (13)	0.06
Lipid-lowering medication, n (%) ^a^	74 (6.9)	51 (9.6)	22 (4.1)	< 0.001
Previous CVD, n (%) ^a^	19 (1.8)	11 (2.1)	8 (1.5)	0.5
*Biochemical analyses*				
Total cholesterol, mmol/L	5.4 (1.4)	5.4 (1.5)	5.4 (1.4)	0.048
Triglycerides, mmol/L	1.0 (0.74)	1.1 (0.71)	0.96 (0.68)	< 0.001
HDL cholesterol, mmol/L	1.6 (0.6)	1.5 (0.6)	1.7 (0.7)	< 0.001
LDL cholesterol, mmol/L	3.2 (1.2)	3.3 (1.0)	3.2 (1.3)	0.9
Creatinine, µmol/L	81 (18)	82 (19)	79 (20)	< 0.001
eGFR, mL/min/1.73 m2	74 (12)	74 (12)	75 (12)	1.0
Glucose, mmol/L	5.6 (0.7)	5.7 (0.8)	5.5 (0.7)	< 0.001
HbA1c, mmol/mol	35 (4)	35 (5)	34 (3)	0.01
CRP, mg/L	0.9 (1.5)	1.0 (1.3)	0.90 (1.4)	0.005
IL-6, pg/mL	1.09 (0.75)	1.06 (0.78)	1.02 (0.75)	0.03
MMP-9, pg/mL	54,700 (37000)	53,600 (38000)	56,200 (37000)	0.04
*Measures of coronary atherosclerosis*,* n (%)*				
Calcium score ≥ 100	117 (11)	67 (13)	49 (9.4)	0.06
Calcium score ≥ 400	36 (3.4)	19 (3.7)	16 (3.1)	0.6
Any stenosis	342 (34)	186 (38)	153 (31)	0.02
Any stenosis ≥ 50%	45 (4.5)	33 (6.7)	12 (2.4)	0.001
Any calcified plaque	319 (32)	172 (35)	145 (29)	0.05
Any non-calcified plaque	39 (3.9)	29 (5.9)	9 (1.8)	< 0.001
SIS > 0	342 (34.1)	186 (37.7)	153 (30.8)	0.02
SIS ≥ 4	78 (7.8)	49 (9.9)	28 (5.6)	0.01

^a^ Data based on questionnaires. Missing values imputed as “no”.

Data presented as median (IQR) for continuous variables and as frequencies (%) for categorical variables. P-values calculated with Mann-Whitney U for group comparison of continuous variables and with the Chi-squared test for categorical variables.

COL1α1 = type I collagen pro-α1 chain; SBP = systolic blood pressure; DBP = diastolic blood pressure; HDL = high density lipoprotein; CRP = C-reactive protein; IL-6 = interleukin-6; LDL = low-density lipoprotein; HbA1c = hemoglobin A1c; eGFR = estimated glomerular filtration rate; SIS = segmental involvement score.

### Relationship between COL1α1 levels and coronary atherosclerosis

**Fig. 1:**
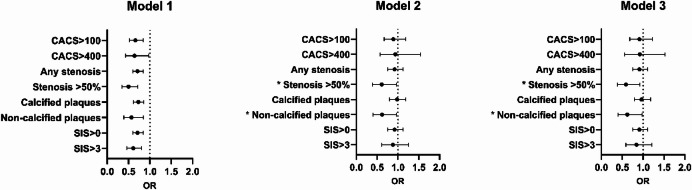
Forest plots showing odds ratios (OR) and corresponding 95% confidence intervals for the association between plasma COL1α1 levels and coronary atherosclerosis unadjusted (*model 1*); adjusted for sex, waist-hip ratio, fasting glucose, high density lipoprotein cholesterol, triglycerides (*model 2*); and for sex, waist hip ratio, age, smoking, the usage of antihypertensive and lipid-lowering medications, diabetes, HbA1c and CRP (*model 3*). COL1α1 levels were analyzed logarithmized with the base of 2. * Indicates remaining statistical significance (*p* < 0.05). CACS = coronary artery calcium score; SIS = segmental involvement score; COL1α1 = type I collagen pro-a1 chain; CRP = C-reactive protein.

In Table 1, the prevalence of coronary atherosclerosis is presented. The low-COL1α1 group exhibited a significantly larger proportion of participants with any coronary stenosis, stenosis ≥ 50%, non-calcified plaques, SIS > 0 and SIS ≥ 4 compared to the high-COL1α1 group. In females, the difference in the prevalence of non-calcified plaques remained significant (Supplementary table S1), whereas for males, this was true for any coronary stenosis ≥ 50% (Supplementary table S2). The associations between COL1α1 levels and coronary atherosclerosis in logistic regression models are presented in in Fig. 1. Lower COL1α1 levels were significantly associated with a higher degree of coronary atherosclerosis, assessed by either CACS or CCTA, in the unadjusted model (model 1, OR_range_=0.50–0.71, *p* < 0.05 for all). After adjusting for sex, waist-hip ratio, fasting glucose, HDL and triglycerides (model 2), COL1α1 remained significantly associated with any stenosis ≥ 50% (OR = 0.61, *p* = 0.03) and non-calcified plaques (OR = 0.62, *p* = 0.03). In model 3, the associations to high grade stenosis (OR = 0.59, *p* = 0.03) and non-calcified plaques (OR = 0.62, *p* = 0.03) remained after adjustment for cardiovascular risk factors. In both model 2 and 3, the associations were mainly attenuated by the introduction of sex as a variable in the models (supplementary table S3 and S4).

## Discussion

The main findings of the study are that COL1α1 in plasma was inversely associated with the presence of coronary atherosclerosis and remained associated with the presence of any coronary stenosis ≥ 50% and non-calcified atherosclerotic plaques after multiple adjustments.

To our knowledge, the findings add to the previously first reported association between plasma COL1α1 and incident coronary events in another Swedish population-based cohort^5^. Furthermore, in line with the reported association to coronary stenosis ≥ 50%, we have previously reported a significant correlation between COL1α1 levels and prevalent angina pectoris^6^. In the present study, we also found an inverse and independent association between COL1α1 and non-calcified atherosclerotic plaques. Interestingly, non-calcified plaques have been linked to a higher risk of future cardiovascular events and appear more similar to histological specimens of vulnerable plaques with thin collagen-deficient fibrous caps^2,22,23^. Recently, a follow-up study of the national SCAPIS cohort (*n* = 24 791) showed that the presence of noncalcified atherosclerosis was associated with a higher risk of coronary events^24^.

In a large number of studies, CRP, IL-6 and MMP-9 have been linked to future coronary events^8,11–13^. Also, MMP-9 together with pro-inflammatory mediators has been shown to be abundantly present in high-risk plaques prone to rupture^11–13,25,26^. The weak associations between inflammatory markers and COL1α1 may indicate that they represent different mechanisms in terms of atherogenesis. In line with this, COL1α1 remained associated with both significant (≥ 50%) coronary stenosis and non-calcified plaques independent of CRP levels. Notably, there is an emerging role of collagen biomarkers in a wide range of cardiovascular conditions such as heart failure, aneurysmal disease and CAD^7,27–29^. Although not fully translatable to this setting, a previous study by our group showcased an association between COL1α1 and PRO-C1 (a type I collagen synthesis marker) but not with C1M (a type I collagen degradation marker). The latter has previously been associated with coronary events^9,30^. As such, COL1α1 builds onto the increasingly recognized role of collagen biomarkers in cardiovascular disease but might represent the less explored synthesis pathway.

When dividing the cohort into a low-COL1α1 and a high-COL1α1 group, it was apparent that COL1α1 levels below the median were associated with several characteristics aligned towards the metabolic syndrome, characterized by abdominal obesity, dyslipidemia, high blood pressure, and fasting glucose^35^. However, as apparent from sex-stratified analysis, some of these findings can be explained by the large COL1α1 difference between sexes. Based on the multivariable models, these factors may partly explain the relationship between circulating COL1α1 and coronary atherosclerosis with sex showing the largest attenuating effect on the associations. Nonetheless, the association with coronary stenosis ≥ 50% and non-calcified plaques remained despite adjustments for these characteristics.

Elaborating on mechanisms, it should be noted that type I collagen is the main constituent of bone tissue, making COL1α1 a plausible proxy for bone turnover^7^. Interestingly, bone turnover has been extensively linked to atherosclerosis, making up the basis for the bone-vascular axis concept^31,32^. Underlining this, one study showcased how a COL1α1 gene polymorphism implicated in osteoporosis was differentially prevalent in myocardial infarction survivors compared to controls^33^. Also, the relationship between insulin resistance, sex and bone health is well-established, where established bone turnover markers are decreased in diabetics and men – aligning with the relationships presented here^34,35^. Lastly, due to the wide flora of functions exerted by type I collagen in atherogenesis, it cannot be ruled out that circulating levels of COL1α1 have causal effects on atherosclerosis, vaguely suggested by positive studies on collagen supplementation and cardiovascular risk factors^36,37^.

Condensing the so far limited evidence on COL1α1 in atherosclerosis, we speculate that low COL1α1 levels could be a marker of a vulnerable plaque phenotype, possibly reflecting the inability to stabilize plaques through type I collagen synthesis^3,4,27^. Also, it is intriguing to speculate that the COL1α1 biomarker represents different mechanisms in the biology of atherosclerosis compared to inflammatory and matrix degradation biomarkers, possibly leading to different plaque phenotypes^3,6,10^. Biomarkers that are associated with non-calcified plaques have the potential to become important clinical complements to non-contrast CT that solely detects calcified plaques, the latter considered a more stable form of atherosclerosis^2,22,23,26^. As discussed, however, several residual confounders could be at play and the exact mechanistic framework for these findings including tissue sources of COL1α1 must be clarified.

One evident limitation of the present study is that it is not possible to link the findings to future events nor infer any causal relationships using a cross-sectional design. Also, being an explorative study with limited statistical power due to few cases with certain coronary characteristics such as high-grade stenosis and non-calcified plaques, we did not correct our analyses for multiple comparisons, increasing the risk for type I errors. The limited prevalence of coronary characteristics also prevented more in-depth exploration using sex-stratified analysis for example. Moreover, data on IL-6 should be interpreted with caution due to high inter-assay coefficients of variation which are probably explained by low concentrations in plasma. The study was conducted in a particular age group in Sweden, making the generalizability to other populations limited.

## Conclusion

Low levels of COL1α1 in plasma, possibly reflecting an impaired type I collagen synthesis, were associated with coronary atherosclerosis, in particular coronary stenosis ≥ 50% and non-calcified atherosclerotic plaques, in a population-based Swedish cohort. These findings suggest that COL1α1 has the potential to be used as a biomarker of coronary atherosclerosis, however, this warrants future studies, ideally involving larger cohorts with higher prevalence of atherosclerotic plaques.

## Supplementary Information

Below is the link to the electronic supplementary material.

Supplementary Material 1

## Data Availability

The data underlying this article are available in the article and raw data can be provided upon reasonable request.
