# Supplementary material for: Circulating type I collagen pro-α1 chain is inversely associated with the presence of coronary atherosclerosis in a Swedish middle-aged population
Source: Sci Rep. 2026 Mar 26;16:9965. doi: 10.1038/s41598-026-45736-2 (PMC13021986; doi:10.1038/s41598-026-45736-2)
Supplement: Supplementary file 1 — Supplementary Material 1 [file 41598_2026_45736_MOESM1_ESM.pdf]

Circulating type I collagen pro-α1 chain is inversely associated with the presence of coronary atherosclerosis in a Swedish middle-aged population

Filip Hammaréus, Lennart Nilsson, Rosanna W.S. Chung, Fredrik H Nyström, Carl Johan Östgren, Lena Jonasson

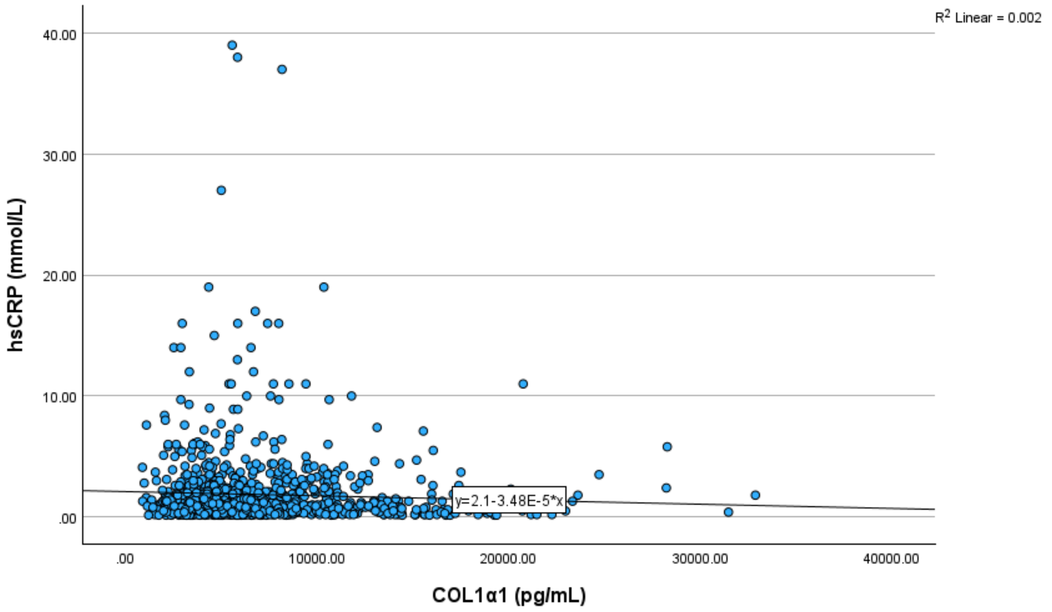

Supplementary figure 1. Scatter plot illustrating the weak negative relationship between type I collagen pro-α1 chain (COL1α1) and high-sensitivity C-reactive protein (hsCRP) plasma concentrations in a Swedish population-based cohort. A linear regression line together with its equation is also presented.

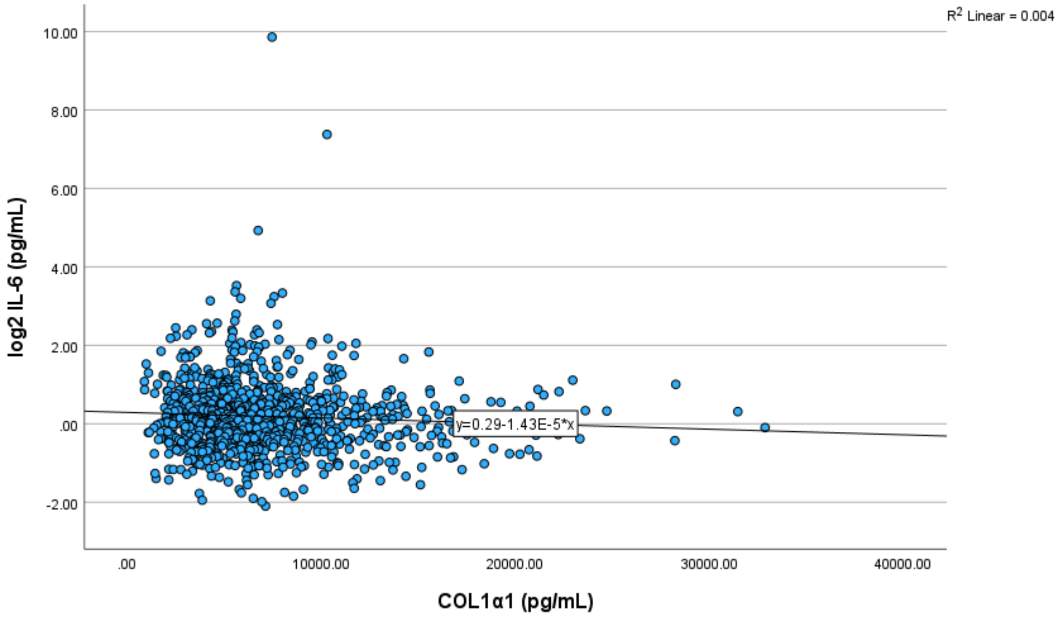

Supplementary figure 2. Scatter plot illustrating the weak negative relationship between type I collagen pro-α1 chain (COL1α1) and $\log_2$ interleukin-6 (IL-6) plasma concentrations in a Swedish population-based cohort. A linear regression line together with its equation is also presented.

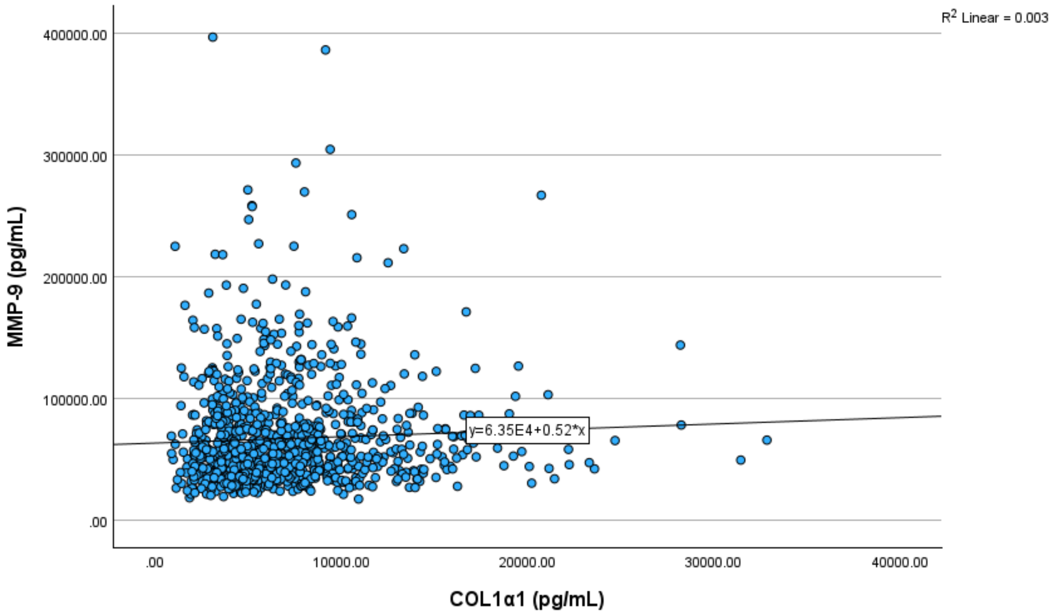

Supplementary figure 3. Scatter plot illustrating the weak positive relationship between type I collagen pro-α1 chain (COL1α1) and matrix metalloproteinase-9 (MMP-9) plasma concentrations in a Swedish population-based cohort. A linear regression line together with its equation is also presented.

Supplementary table S1. Characteristics of all females in the study population and divided by high or low COL1α1 levels (below or above median level)

COL1α1 (pg/mL)	Total (n=535)	Low COL1α1 (n=267)	High COL1α1 (n=268)	*p*
	7300 (5600)	5000 (2400)	10 600 (5300)	<0.001
Characteristics				
Age, years	56 (7.7)	57 (7.9)	56 (7.6)	0.4
Current smoker, n (%)	36 (6.7)	19 (7.2)	17 (6.4)	0.7
Waist-hip ratio [a]	0.85 ± 0.068	0.85 ± 0.068	0.84 ± 0.067	0.02
Diabetes, n (%)	33 (6.2)	25 (9.5)	7 (2.6)	<0.001
Anti-hypertensive medication, n (%) [b]	74 (14)	46 (17)	26 (10)	0.01
Office SBP, mmHg	128 (25)	129 (25)	124 (24)	0.06
Office DBP, mmHg	82 (14)	83 (15)	81 (13)	0.09
Lipid-lowering medication, n (%) [b]	26 (4.9)	20 (7.6)	5 (1.9)	0.002
Previous CVD, n (%) [a]	2 (0.4)	1 (0.4)	1 (0.4)	1
Biochemical analyses				
Total cholesterol, mmol/L	5.6 (1.4)	5.6 (1.3)	5.6 (1.5)	0.4
Triglycerides, mmol/L	0.95 (0.59)	1.0 (0.64)	0.85 (0.54)	<0.001
HDL cholesterol, mmol/L	1.8 (0.7)	1.7 (0.6)	1.9 (0.6)	<0.001
LDL cholesterol, mmol/L	3.2 (1.2)	3.2 (1.2)	3.2 (1.3)	0.9
Creatinine, µmol/L	72 (14)	72 (14)	72 (13)	0.8
eGFR [a]	75 ± 9.7	75 ± 10	75 ± 9.3	0.8
Glucose, mmol/L	5.5 (0.8)	5.6 (0.8)	5.4 (0.6)	<0.001
HbA1c, mmol/mol	35 (4.0)	35 (4.0)	35 (3.0)	0.09
CRP, mg/L	1.0 (1.6)	1.1 (1.5)	0.8 (1.5)	0.009
IL-6, pg/mL	1.1 (0.79)	1.1 (0.92)	1.0 (0.74)	0.02
MMP-9, pg/mL	54 700 (38 000)	51 000 (36 000)	59 300 (36 000)	0.001
Measures of coronary atherosclerosis, n (%)				
Calcium score ≥100	24 (4.5)	15 (5.8)	9 (3.4)	0.3
Calcium score ≥400	6 (1.1)	4 (1.5)	2 (0.8)	0.6
Any stenosis	108 (22)	57 (24)	50 (20)	0.3
Any stenosis ≥50%	6 (1.2)	4 (1.7)	2 (0.8)	0.4
Any calcified plaque	95 (19)	48 (20)	47 (19)	0.7
Any non-calcified plaque	13 (2.6)	10 (4.2)	2 (0.8)	0.02
SIS > 0	108 (22)	57 (24)	50 (20)	0.3
SIS ≥ 4	12 (2.4)	8 (3.3)	4 (1.6)	0.2

[a] Normally distributed and thus presented as mean ± SD and p-value calculated with t-test

[b] Data based on questionnaires. Missing values imputed as “no”

Data presented as median (IQR) for continuous variables and as frequencies (%) for categorical variables. P-values calculated with Mann-Whitney U for group comparison of continuous variables and with the Chi-squared test for categorical variables.

COL1α1 = type I collagen pro-α1 chain; SBP = systolic blood pressure; DBP = diastolic blood pressure; HDL = high density lipoprotein; CRP = C-reactive protein; IL-6 = interleukin-6; MMP-9 = matrix metalloproteinase-9; LDL = low-density lipoprotein; HbA1c = hemoglobin A1c; eGFR = estimated glomerular filtration rate; SIS = segmental involvement score

**Supplementary table S2.** Characteristics of all males in the study population and divided by high or low COL1α1 levels (below or above median level)				
COL1α1 (pg/mL)	Total (n=543)	Low COL1α1 (n=271)	High COL1α1 (n=272)	*p*
	5200 (3300)	3800 (1500)	7400 (2500)	<0.001
Characteristics				
Age, years	58 (7.3)	58 (7.3)	58 (7.8)	0.2
Current smoker, n (%)	39 (7.2)	23 (8.6)	15 (5.6)	0.2
Waist-hip ratio	0.96 (0.09)	0.97 (0.08)	0.96 (0.09)	0.04
Diabetes, n (%)	53 (9.8)	37 (13.9)	14 (5.2)	<0.001
Anti-hypertensive medication, n (%) [a]	116 (21)	62 (23)	52 (20)	0.3
Office SBP, mmHg	132 (21)	133 (24)	131 (20)	0.2
Office DBP, mmHg	83 (13)	83 (13)	82 (13)	0.3
Lipid-lowering medication, n (%) [a]	48 (8.8)	30 (11)	18 (6.7)	0.07
Previous CVD, n (%) [a]	9 (1.7)	6 (2.2)	3 (1.1)	0.3
Biochemical analyses				
Total cholesterol, mmol/L	5.2 (1.4)	5.3 (1.6)	5.2 (1.3)	0.7
Triglycerides, mmol/L	1.2 (0.86)	1.3 (0.89)	1.1 (0.70)	<0.001
HDL cholesterol, mmol/L	1.4 (0.5)	1.3 (0.5)	1.4 (0.5)	0.06
LDL cholesterol, mmol/L	3.2 (1.3)	3.2 (1.5)	3.2 (1.0)	0.8
Creatinine, µmol/L	89 (15)	88 (15)	89 (15)	0.09
eGFR	74 (11)	75 (11)	74 (12)	0.2
Glucose, mmol/L	5.7 (0.7)	5.8 (0.9)	5.6 (0.6)	<0.001
HbA1c, mmol/mol	35 (4.0)	35 (5.0)	43 (4.0)	0.08
CRP, mg/L	0.9 (1.4)	1.0 (1.4)	0.8 (1.4)	0.08
IL-6, pg/mL	1.1 (0.72)	1.1 (0.75)	1.1 (0.69)	0.07
MMP-9, pg/mL	54 700 (36 800)	54 400 (36 900)	55 100 (36 100)	0.5
Measures of coronary atherosclerosis, n (%)				
Calcium score ≥100	93 (18)	50 (20)	42 (16)	0.5
Calcium score ≥400	30 (5.8)	17 (6.7)	12 (4.7)	0.5
Any stenosis	234 (46)	117 (47)	115 (46)	0.7
Any stenosis ≥50%	39 (7.7)	28 (11)	11 (4.3)	0.004
Any calcified plaque	224 (44)	112 (45)	110 (44)	0.7
Any non-calcified plaque	26 (5.1)	17 (6.8)	9 (3.6)	0.1
SIS > 0	234 (46)	117 (47)	115 (46)	0.7
SIS ≥ 4	66 (13)	38 (15)	27 (11)	0.1

[a] Data based on questionnaires. Missing values imputed as “no”

Data presented as median (IQR) for continuous variables and as frequencies (%) for categorical variables. P-values calculated with Mann-Whitney U for group comparison of continuous variables and with the Chi-squared test for categorical variables.

COL1α1 = type I collagen pro-α1 chain; SBP = systolic blood pressure; DBP = diastolic blood pressure; HDL = high density lipoprotein; CRP = C-reactive protein; IL-6 = interleukin-6; MMP-9 = matrix metalloproteinase-9; LDL = low-density lipoprotein; HbA1c = hemoglobin A1c; eGFR = estimated glomerular filtration rate; SIS = segmental involvement score

**Supplementary table S3.** Model 1 (univariable) and model 2 (multivariable stepwise) logistic regression models for associations between plasma COL1α1 and different measures of coronary atherosclerosis assessed on non-contrast and contrast-enhanced computed tomography scans		
Measures of coronary atherosclerosis	OR (95% CI)	p-value
Model 1		
Calcium score ≥100	0.66 (0.52–0.84)	<0.001
Calcium score ≥400	0.64 (0.43–0.97)	0.04
Any stenosis	0.71 (0.60–0.84)	<0.001
Any stenosis ≥50%	0.50 (0.35–0.72)	<0.001
Any calcified plaque	0.73 (0.61–0.86)	<0.001
Any non-calcified plaque	0.57 (0.39–0.85)	0.006
SIS > 0	0.71 (0.60–0.84)	<0.001
SIS ≥ 4	0.61 (0.46–0.81)	<0.001
Model 2 – sex		
Calcium score ≥100	0.80 (0.61–1.1)	0.1
Calcium score ≥400	0.77 (0.48–1.2)	0.3
Any stenosis	0.84 (0.70–1.0)	0.06
Any stenosis ≥50%	0.58 (0.36–0.85)	0.007
Any calcified plaque	0.88 (0.73–1.1)	0.2
Any non-calcified plaque	0.61 (0.40–0.93)	0.02
SIS > 0	0.80 (0.61–1.1)	0.06
SIS ≥ 4	0.77 (0.48–1.2)	0.06
Model 2 – sex, WHR		
Calcium score ≥100	0.86 (0.65–1.1)	0.3
Calcium score ≥400	0.84 (0.52–1.4)	0.5
Any stenosis	0.89 (0.74–1.1)	0.2
Any stenosis ≥50%	0.56 (0.37–0.87)	0.009
Any calcified plaque	0.93 (0.77–1.1)	0.4
Any non-calcified plaque	0.60 (0.39–0.93)	0.02
SIS > 0	0.89 (0.74–1.1)	0.2
SIS ≥ 4	0.79 (0.56–1.1)	0.2
Model 2 - sex, WHR, f-glucose		
Calcium score ≥100	0.90 (0.68–1.2)	0.5
Calcium score ≥400	0.93 (0.57–1.5)	0.8
Any stenosis	0.93 (0.77–1.1)	0.5
Any stenosis ≥50%	0.61 (0.39–0.95)	0.03
Any calcified plaque	0.98 (0.81–1.2)	0.9
Any non-calcified plaque	0.62 (0.40–0.96)	0.03
SIS > 0	0.93 (0.77–1.1)	0.5
SIS ≥ 4	0.86 (0.61–1.2)	0.4
Model 2 - sex, WHR, f-glucose, HDL-C		
Calcium score ≥100	0.89 (0.67–1.2)	0.4
Calcium score ≥400	0.93 (0.57–1.5)	0.8
Any stenosis	0.92 (0.76–1.1)	0.4
Any stenosis ≥50%	0.61 (0.39–0.95)	0.03
Any calcified plaque	0.98 (0.80–1.2)	0.8
Any non-calcified plaque	0.62 (0.40–0.96)	0.03
SIS > 0	0.92 (0.76–1.1)	0.4
SIS ≥ 4	0.87 (0.61–1.2)	0.4
Model 2 - sex, WHR, f-glucose, HDL-C, TG		
Calcium score ≥100	0.89 (0.67–1.2)	0.4
Calcium score ≥400	0.94 (0.57–1.5)	0.8
Any stenosis	0.92 (0.76–1.1)	0.4
Any stenosis ≥50%	0.61 (0.39–0.96)	0.03
Any calcified plaque	0.98 (0.80–1.2)	0.8
Any non-calcified plaque	0.62 (0.40–0.96)	0.03
SIS > 0	0.92 (0.76–1.1)	0.4
SIS ≥ 4	0.88 (0.61–1.2)	0.5

Model 1 unadjusted; model 2 adjusted for sex, waist-hip ratio (WHR), fasting glucose (f-glucose), high density lipoprotein cholesterol (HDL-C) and triglycerides (TG); COL1α1 levels were analyzed logarithmized with the base of 2.

COL1α1 = type I collagen pro-a1 chain; OR = odds ratio; SIS = segmental involvement score; CRP = C-reactive protein

**Supplementary table S4.** Model 1 (univariable) and model 3 (multivariable stepwise) logistic regression models for associations between plasma COL1α1 and different measures of coronary atherosclerosis assessed on non-contrast and contrast-enhanced computed tomography scans		
Measures of coronary atherosclerosis	OR (95% CI)	p-value
Model 1		
Calcium score ≥100	0.66 (0.52–0.84)	<0.001
Calcium score ≥400	0.64 (0.43–0.97)	0.04
Any stenosis	0.71 (0.60–0.84)	<0.001
Any stenosis ≥50%	0.50 (0.35–0.72)	<0.001
Any calcified plaque	0.73 (0.61–0.86)	<0.001
Any non-calcified plaque	0.57 (0.39–0.85)	0.006
SIS > 0	0.71 (0.60–0.84)	<0.001
SIS ≥ 4	0.61 (0.46–0.81)	<0.001
Model 3 – sex		
Calcium score ≥100	0.80 (0.61–1.1)	0.1
Calcium score ≥400	0.77 (0.48–1.2)	0.3
Any stenosis	0.84 (0.70–1.0)	0.06
Any stenosis ≥50%	0.58 (0.36–0.85)	0.007
Any calcified plaque	0.88 (0.73–1.1)	0.2
Any non-calcified plaque	0.61 (0.40–0.93)	0.02
SIS > 0	0.80 (0.61–1.1)	0.06
SIS ≥ 4	0.77 (0.48–1.2)	0.06
Model 3 – sex, WHR		
Calcium score ≥100	0.86 (0.65–1.1)	0.3
Calcium score ≥400	0.84 (0.52–1.4)	0.5
Any stenosis	0.89 (0.74–1.1)	0.2
Any stenosis ≥50%	0.56 (0.37–0.87)	0.009
Any calcified plaque	0.93 (0.77–1.1)	0.4
Any non-calcified plaque	0.60 (0.39–0.93)	0.02
SIS > 0	0.89 (0.74–1.1)	0.2
SIS ≥ 4	0.79 (0.56–1.1)	0.2
Model 3 – sex, WHR, age		
Calcium score ≥100	0.87 (0.66–1.2)	0.3
Calcium score ≥400	0.87 (0.54–1.4)	0.6
Any stenosis	0.90 (0.74–1.1)	0.3
Any stenosis ≥50%	0.58 (0.37–0.89)	0.01
Any calcified plaque	0.94 (0.77–1.1)	0.6
Any non-calcified plaque	0.61 (0.39–0.93)	0.02
SIS > 0	0.90 (0.74–1.1)	0.3
SIS ≥ 4	0.81 (0.57–1.1)	0.2
Model 3 – sex, WHR, age, smoking		
Calcium score ≥100	0.88 (0.66–1.2)	0.4
Calcium score ≥400	0.88 (0.54–1.4)	0.6
Any stenosis	0.90 (0.74–1.1)	0.3
Any stenosis ≥50%	0.58 (0.37–0.90)	0.02
Any calcified plaque	0.95 (0.78–1.2)	0.6
Any non-calcified plaque	0.60 (0.39–0.92)	0.02
SIS > 0	0.90 (0.74–1.1)	0.3
SIS ≥ 4	0.81 (0.58–1.1)	0.2
Model 3 – sex, WHR, age, smoking, HT meds		
Calcium score ≥100	0.89 (0.67–1.2)	0.4
Calcium score ≥400	0.88 (0.54–1.4)	0.6
Any stenosis	0.91 (0.75–1.1)	0.3
Any stenosis ≥50%	0.58 (0.38–0.90)	0.02
Any calcified plaque	0.95 (0.78–1.2)	0.6
Any non-calcified plaque	0.60 (0.39–0.92)	0.02

SIS > 0	0.91 (0.75–1.1)	0.3
SIS ≥ 4	0.82 (0.58–1.2)	0.3
Model 3 – sex, WHR, age smoking, HT meds, lipid meds		
Calcium score ≥100	0.91 (0.68–1.2)	0.5
Calcium score ≥400	0.95 (0.58–1.6)	0.8
Any stenosis	0.91 (0.75–1.1)	0.3
Any stenosis ≥50%	0.58 (0.37–0.90)	0.02
Any calcified plaque	0.96 (0.78–1.2)	0.7
Any non-calcified plaque	0.58 (0.38–0.90)	0.02
SIS > 0	0.91 (0.75–1.1)	0.3
SIS ≥ 4	0.83 (0.59–1.2)	0.3
Model 3 – sex, WHR, age, smoking, HT meds, lipid meds, diabetes		
Calcium score ≥100	0.91 (0.68–1.2)	0.5
Calcium score ≥400	0.92 (0.56–1.5)	0.8
Any stenosis	0.91 (0.75–1.1)	0.4
Any stenosis ≥50%	0.59 (0.38–0.92)	0.02
Any calcified plaque	0.96 (0.79–1.2)	0.7
Any non-calcified plaque	0.62 (0.40–0.96)	0.03
SIS > 0	0.91 (0.75–1.1)	0.4
SIS ≥ 4	0.84 (0.59–1.2)	0.4
Model 3 – sex, WHR, age, smoking, HT meds, lipid meds, diabetes, HbA1c		
Calcium score ≥100	0.92 (0.68-1.2)	0.6
Calcium score ≥400	0.97 (0.58-1.6)	0.9
Any stenosis	0.90 (0.74-1.1)	0.3
Any stenosis ≥50%	0.59 (0.37-0.95)	0.03
Any calcified plaque	0.97 (0.79-1.2)	0.7
Any non-calcified plaque	0.62 (0.40-0.96)	0.03
SIS > 0	0.91 (0.75-1.1)	0.4
SIS ≥ 4	0.85 (0.59-1.2)	0.4
Model 3 – sex, WHR, age, smoking, HT meds, lipid meds, diabetes, HbA1c, CRP		
Calcium score ≥100	0.92 (0.68-1.2)	0.6
Calcium score ≥400	0.97 (0.58-1.6)	0.9
Any stenosis	0.90 (0.74-1.1)	0.3
Any stenosis ≥50%	0.59 (0.37-0.95)	0.03
Any calcified plaque	0.97 (0.79-1.2)	0.7
Any non-calcified plaque	0.62 (0.40-0.97)	0.03
SIS > 0	0.91 (0.75-1.1)	0.4
SIS ≥ 4	0.85 (0.60-1.2)	0.4

Model 1 unadjusted; model 3 adjusted for independent cardiovascular risk factors including sex, waist-hip ratio (WHR), age, smoking, antihypertensive medications (HT meds), lipid-lowering medications (lipid meds), diabetes, Hemoglobin A1C (HbA1c) and c-reactive protein (CRP). COL1α1 levels were analyzed logarithmized with the base of 2.

COL1α1 = type I collagen pro-a1 chain; OR = odds ratio; SIS = segmental involvement score
